# Performance of an Annular Closure Device in a ‘Real-World’, Heterogeneous, At-Risk, Lumbar Discectomy Population

**DOI:** 10.7759/cureus.1824

**Published:** 2017-11-06

**Authors:** Adisa Kuršumović, Stefan Rath

**Affiliations:** 1 Neurosurgery, Spinal Surgery, and Interventional Neuroradiology, Donauisar Klinikum Deggendorf

**Keywords:** lumbar, disc, discectomy, limited discectomy, herniation, reherniation, recurrent herniation, annular closure, annular defect, registry

## Abstract

Study design/setting

Retrospective analysis of single-center registry outcomes data.

Objective

Assess the utility of an annular closure device (ACD) as an adjunct to limited discectomy for lumbar disc herniation (LDH).

Background

Recurrent lumbar disc herniation (rLDH) following limited discectomy persists at clinically significant rates, especially in large annular defect (at least 6 mm width) patients. While the etiology of reherniation is often multifactorial, inadequate annular occlusion remains one of the foremost considerations. Accordingly, annular closure has emerged as a promising technique and is the focus of this analysis.

Methods

This was a retrospective analysis of 171 patients who underwent limited lumbar discectomy with an ACD for LDH. Standardized patient assessment was performed preoperatively, three months postoperatively, and 12 months postoperatively, in addition to self-presented visits. No minimum last follow-up was required for inclusion. Oswestry Disability Index (ODI) and Visual Analog Scale (VAS Leg/Back) pain scores were collected at all visits. Plain radiographs were obtained at all visits, with magnetic resonance imaging (MRI) scans performed annually and/or when patients presented as symptomatic. ACD-related complications due to partial or complete mesh detachment from the titanium anchor were reported. All secondary surgical interventions were also reported. The Wilcoxon Rank-sum test was used to compare outcomes and events between sub-groups (p < 0.05).

Results

Mean last follow-up for all patients was 15 months. Large annular defects were present in 154 patients (90%). Symptomatic reherniations were observed in six patients (3.5%; five were present in the large annular defect subpopulation). All patients demonstrated clinically meaningful improvement in clinical outcome scores at both follow-up intervals. ACD mesh detachment was observed in 15 patients (8.8%; two underwent a subsequent surgical intervention). No symptomatic reherniations were observed in secondary herniation patients compared to six (4.1%) in the primary herniation group (p = 0.60).

Conclusions

Annular closure with the ACD results in clinically meaningful improvements in both primary and secondary LDH patients, with decreased rates of reherniation in high-risk patients compared to previous discectomy reports.

## Introduction

Of all spinal procedures performed, lumbar discectomy for disc herniation is the most common, with approximately 300,000 annual procedures in the United States alone [1-3]. While the majority of patients experience significant early-postoperative reduction in symptoms following primary discectomy, incidence of same-level symptomatic reherniation requiring reoperation has been reported at rates ranging from 3% to 18.9% [4-15]. In addition to the risks associated with exposing the patient to another surgery, the economic burden of treating symptomatic reherniation is substantial, with direct and indirect costs estimated at $34,242 and $3,778, respectively [1, 16]. Furthermore, these patients are at considerable risk for additional, ancillary, spinal surgery, with the tendency to achieve poorer results with each subsequent intervention [8, 11, 17, 18].

In light of these trends, efforts have been made to better characterize associated risk factors that may predispose discectomy patients to herniation recurrence. One of the most pronounced at-risk subpopulations includes patients with large annular defects (at least 6 mm) where rates of symptomatic recurrence have been reported to range from 18.0% to 27.3% following limited discectomy [19, 20]. While a more aggressive (subtotal) discectomy can be advantageous in preventing recurrence in this subgroup, greater removal of disc material is also associated with disc height loss and a subsequent cascade of comorbidities, such as increased pain and disability [12, 21-25]. Hence, a paradox exists in which limited disc removal can simultaneously result in better patient satisfaction than a subtotal approach while increasing the risk of recurrence [4]. Therefore, the ability to provide more effective annular closure in this large defect subgroup is critical.

A bone-anchored annular closure device has been designed and developed with the intent to address this treatment gap. The annular closure device (ACD) mimics the structure of healthy annulus, creating a barrier for the conserved nucleus material to retain it within the disc space. Initial reported outcomes with the ACD have been promising. A prospective study by Parker, et al., comparing primary limited discectomy outcomes with and without ACD, reported 12-month index-level reherniation rates of 0% (ACD) and 6.5% (No ACD), respectively [22]. Similarly, Bouma, et al., conducting a prospective study of the ACD in primary discectomy patients, reported a symptomatic reherniation rate of 1.45% in large defect (at least 6 mm wide) subjects (Mean follow-up: 18.7 months) [26].

However, despite these encouraging early prospective cohort outcomes, there remains a need for robust prospective, randomized controlled trial (RCT) evidence and confirmatory registry data for establishing ACD as a solution for annular closure. The combination of RCT evidence supported by registry evidence is a respected approach for the widespread adoption of novel technologies [27].

In the case of the aforementioned ACD, an RCT is currently on-going, assessing outcomes specifically in large defect, limited discectomy patients (Clinicaltrials.gov ID: NCT01283438). The goal of this study is to report outcomes with the ACD in a more heterogeneous ‘real-world’ population (i.e., variation in implant size; both primary & secondary discectomy, lack of six-week conservative treatment prior to operation) for comparative purposes with historical prospective data and pending RCT data.

## Materials and methods

Study design and data collection

**Figure 1:**
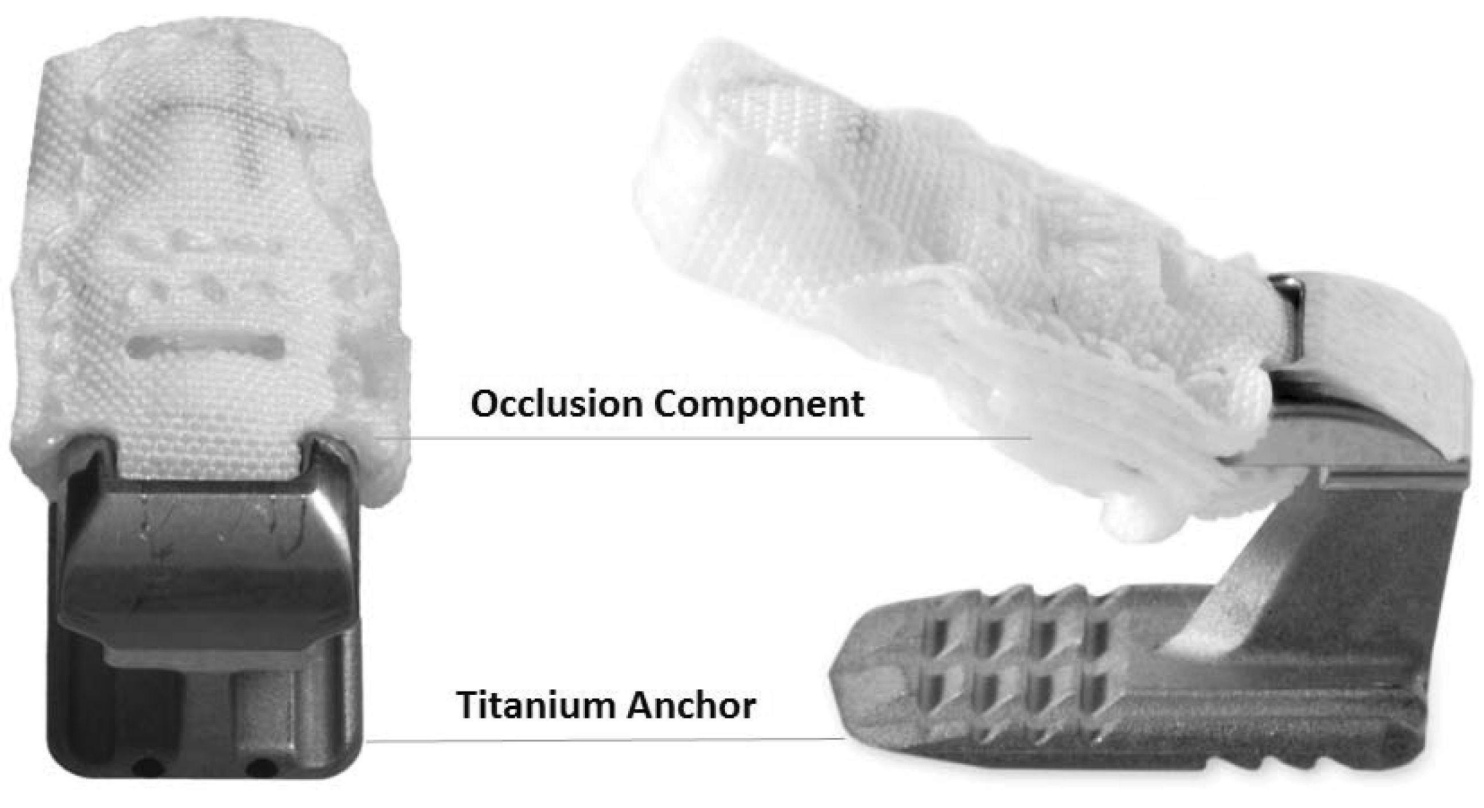
Annular closure device possessing a mesh occlusion component and titanium anchor.

**Figure 2:**
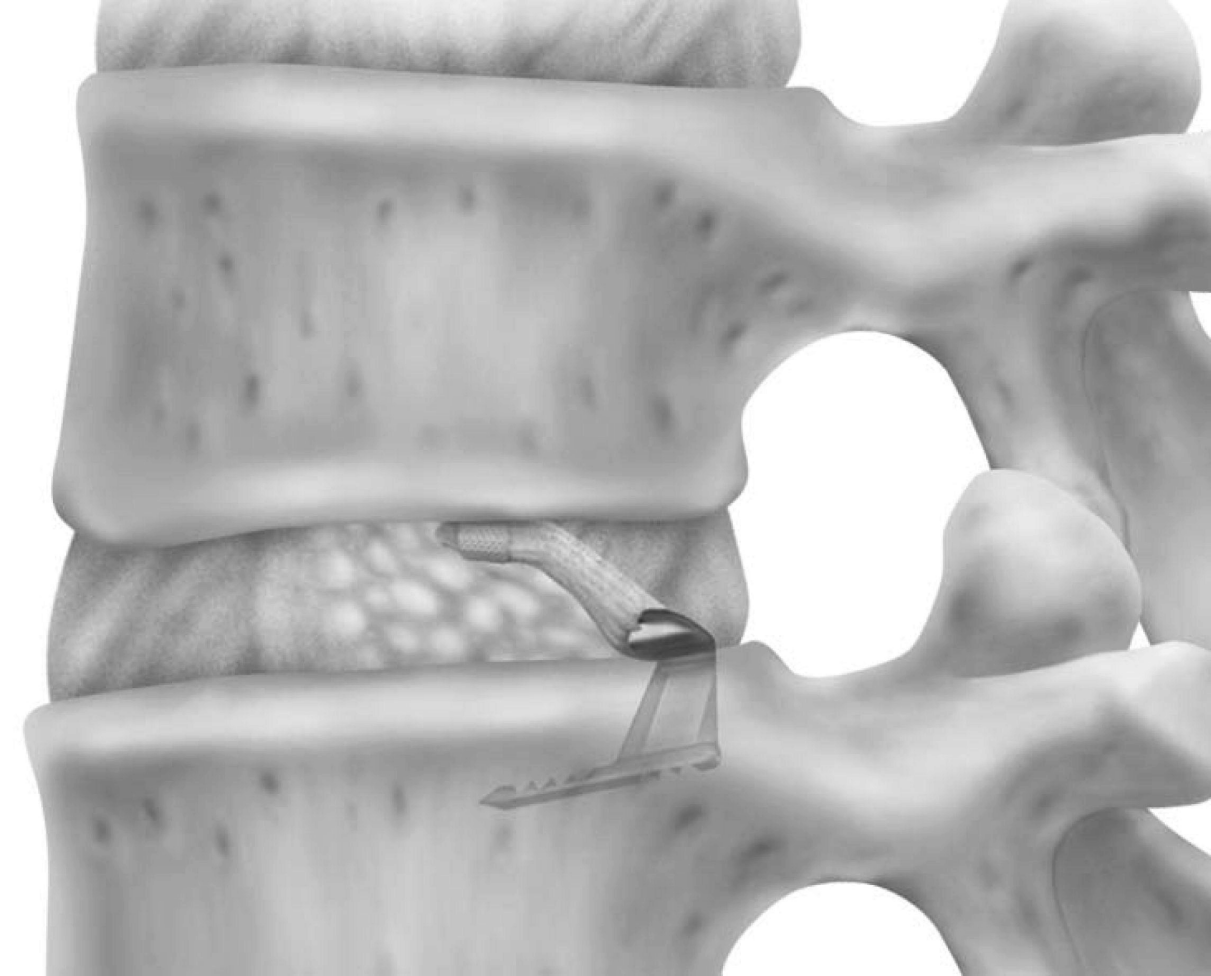
Rendering of the annular closure device following implantation.

This was a single-center registry retrospective study of 171 patients who underwent limited lumbar discectomy with an ACD (Barricaid, Intrinsic Therapeutics, Woburn, MA) (Figures 1, 2). The study protocol was approved by the local medical ethics committee. All clinical outcomes and patient imaging had been collected previously in accordance with institutional standard of care. All procedures were performed between September 2009 and September 2015 by one of 17 operating surgeons (eight residents and nine board-certified neurosurgeons), all of whom were experienced in both limited discectomy and ACD techniques.

Patient selection

Patients who met the following criteria were included in the study: (1) diagnosis of herniated or reherniated disc with sciatica accompanied by radicular symptoms, (2) preoperative magnetic resonance imaging (MRI) or computed tomography (CT) confirming disc herniation, and (3) must have had available: preoperative and postoperative Visual Analog Scale (VAS Back/Leg) and Oswestry Disability Index (ODI) scores, recorded size of the annular defect, and recorded size of the ACD used to close the defect.

Surgical technique

All patients received prophylactic antibiotics before skin incision. A standard posterior lumbar limited discectomy was performed and, where possible, the discectomy was carried out through the interlaminar space alone. In cases where the interlaminar space alone did not provide sufficient access, a small unilateral laminotomy was performed. Medial facetectomy was done only when impingement of the nerve root by the medial facet was noted.

After completion of the limited discectomy, the height and width of the annular defect was measured using designated defect measurement tools. A sizing trial, designed to replicate the size and shape of the loaded ACD delivery tool, was then used to confirm access through the lamina and determine the appropriate angle of approach to the disc. The implant was placed in the disc space with the delivery tool under fluoroscopic control, ensuring that the angle of approach was parallel to the target endplate in the region of implantation. The ACD’s anchor was impacted into the endplate, which simultaneously placed the mesh in position within the disc.

Postoperative imaging was performed one to two days after surgery to confirm the position of the ACD. Patients were mobilized immediately following the operation and discharged without restrictions. Physical therapy was ordered if neurologic deficit was observed.

Annular closure device

**Figure 3:**
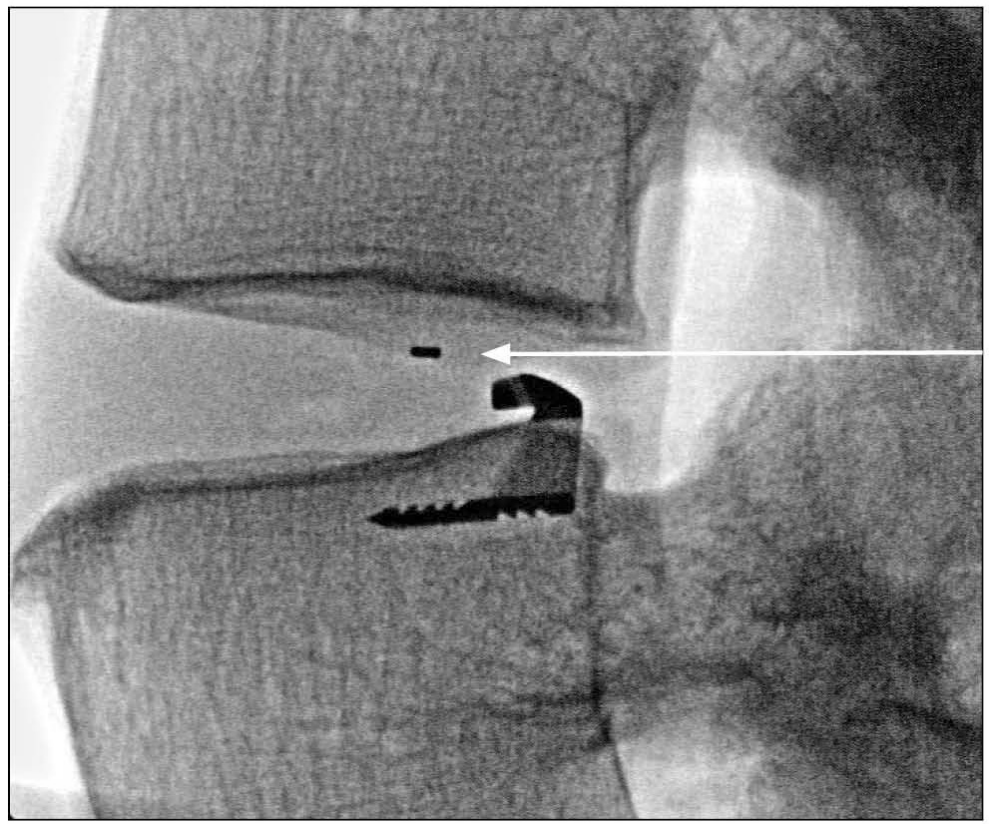
Postoperative plain radiograph showing platinum-iridium marker (arrow) within the annular closure device polymer mesh.

The ACD under consideration in this study is intended for use as an adjunct to lumbar discectomy surgery (Figures 1, 2). It is designed to block large annular defects following a sequestrectomy or limited discectomy, intending to prevent extrusion of the nucleus material from within the disc space. The device is composed of two components, a flexible mesh that blocks the defect and inhibits nucleus extrusion (the mesh overlies the residual nucleus) and a bone anchor that secures the mesh component to one of the adjacent vertebral bodies (Figure 1). The mesh material is woven polyester. A platinum-iridium marker within the polymer mesh allows visualization on plain radiographs (Figure 3). The bone anchor is composed of titanium alloy (Ti-6Al-4V Extra Low Interstitial). The anchor is placed into one of the adjacent vertebral bodies via tamp and mallet. The implant is available in three mesh widths (8 mm, 10 mm and 12 mm) and is provided pre-loaded on a disposable delivery tool.

Study follow-up and outcome measures

Data were collected preoperatively and routine postoperative follow-ups included visits at three months and annually thereafter in addition to any unscheduled visits where patients self-presented. ODI and VAS (Back/Leg) scores were collected preoperatively and at each follow-up visit. Plain radiographs and functional imaging (MRI or CT) were obtained preoperatively, with radiographs performed again at all postoperative follow-up visits. MRI was obtained at annual visits and at any visit in which pain was reported. Reasons for preoperative CT instead of MRI included contraindications or long waiting lists for MRI, with the large majority receiving MRI.

Ipsilateral and contralateral reherniations were identified via functional imaging and reported as adverse events. Symptomatic reherniation was confirmed if any protrusion, extrusion, or sequestration of disc material were present accompanied by radicular symptoms. All functional imaging was reviewed by one spine surgeon experienced in using the ACD. Complications associated with either partial or complete detachment of the ACD's mesh from its titanium anchor were reviewed and separately analyzed. All other types of reoperations and complications occurring within the follow-up period were also reported.

Statistical analysis

The Wilcoxon Rank-sum test was used to compare clinical outcomes scores and rate of adverse events between population sub-groups (i.e., primary vs. secondary discectomy; mesh dislocation vs. no dislocation). Findings were deemed to be statistically significant where p ≤ 0.05.

## Results

Patient demographics

A total of 171 patients met the screening criteria, all receiving limited discectomy with the ACD for either a primary (n = 145) or secondary (n = 26) herniation. Of the total population, 154 patients (90%) presented with a large annular defect (at least 6 mm width). The mean age for all patients was 45.7 years (range 18 to 75; Table 1). There were 69 patients (40.4%) implanted with an 8 mm ACD, 91 (53.2%) implanted with a 10 mm ACD, and 11 (6.4%) implanted with a 12 mm ACD.

**Table 1:** Study population demographics. L2-3: Disc between the second and third lumbar vertebrae L3-4: Disc between the third and fourth lumbar vertebrae L4-5: Disc between the fourth and fifth lumbar vertebrae L5-6: Disc between the fifth and sixth lumbar vertebrae (one patient had a sixth lumbar vertebra) L5-S1: Disc between the fifth lumbar vertebra and first sacral vertebra

Gender (Male : Female)	97M : 74F
Age (years)	45.7 (SD 14.0)
Operative Level (n)	
L2-3	1
L3-4	6
L4-5	98
L5-6	1
L5-S1	64
Defect Area (mm^2^)	42.8 (SD 12.9)
% with defect width > 6 mm	90% (154/171)
SD: Standard Deviation	

Follow-up

The mean latest follow-up for all 171 patients was 15 months (range: 1-71.8 months). Per the intended follow-up schedule, clinical scores were reported for 130 patients (76%) within the first three months postoperatively and 127 patients (74%) at 12 months or later.

Herniation recurrence

A total of six symptomatic reherniations (3.5%) were observed. Of those, five reherniations occurred within the first two months after implantation, with the remaining event occurring at nine months. There were four recurrences identified on the ipsilateral side and treated by surgical intervention, yielding a reoperative recurrence rate of 2.3%. There were two reherniations on the contralateral side and were treated conservatively. Within the large annular defect patient subset, there were five symptomatic reherniations (3.2%).

Reherniations without radicular symptoms were observed via imaging in five patients from the total population (2.9%). Of those five patients, two were completely asymptomatic, while the remaining three reported back pain only and were treated with facet joint infiltrations. The asymptomatic reherniations both occurred within the first nine months, while the reherniations with back pain, but no radicular symptoms, occurred after 16 months.

Patient-reported outcomes

All patients demonstrated clinically meaningful improvement in clinical outcome scores (ODI, VAS Leg/Back) at both the early (less than three months) and later (at least 12 months) time intervals, even when stratified by primary/secondary reherniation. Scores are summarized in Table 2.

**Table 2:** Preoperative and postoperative patient reported outcome scores. ODI: Oswestry Disability Index; VAS: Visual Analog Scale for pain.

Preoperative (n = 171*)	
ODI	53.0 ± 20.9
VAS Leg	79.1 ± 23.3
VAS Back	58.9 ± 31.2
≤ 3 Months (n = 130)	
ODI	19.9 ± 17.6
VAS Leg	20.5 ± 23.9
VAS Back	24.5 ± 20.1
12+ Months (n = 127*)	
ODI	15.8 ± 16.9
VAS Leg	23.3 ± 27.1
VAS Back	26.9 ± 24.8
Values represented as: Mean ± Standard Deviation	
*One patient reported ODI only.	

Subsequent surgical interventions

A total of 22 subsequent surgical interventions were performed across 12 subjects (7%). There were 13 procedures performed within 12 months post-op, seven between 12 and 24 months, and two between 24 and 36 months. There were three patients who were reoperated outside of the investigating institution, so details of the reoperations were not obtainable. Subsequent discectomies were performed in four patients following symptomatic reherniations, including removal of the ACD from one patient. The remaining reoperations are summarized in Table 3, including a series of wound revision procedures in a single subject (n = 4), decompression due to early epidural hematoma and new paresis (n = 1), decompression and aggressive discectomy to remove granulation tissue that was compressing the nerve root (ACD removed) (n = 1), and fusion to address disc degeneration and/or collapse (n = 2).

**Table 3:** Summary of reoperations.

	Number of Procedures	Number of Subjects (%)
Fusion	5	5 (2.9%)
Discectomy	5	5 (2.9%)
Wound Revision	4	1 (0.6%)
Decompression	3	3 (1.8%)
Unknown (performed outside of institution)	3	3 (1.8%)
Other (spinal cord stimulator)	2	1 (0.6%)

ACD complications and radiographic outcomes

The ACD's mesh partially or completely detached from its titanium anchor in 15 patients (8.8%). However, only two of these patients underwent a subsequent surgical intervention. In three of the 15 mesh detachments (20.0%), the mesh had penetrated the endplate of the vertebral body, but remained within the disc space. Average time before identification of mesh detachment was 25.9 months (range 3.5 to 57.7). VAS Back and ODI scores in patients with mesh detachment were statistically significantly inferior to patients without detachment by 12 months (p ≤ 0.04; Table 4).

**Table 4:** Postoperative pain and function scores in patients with and without annular closure device (ACD) mesh detachments. ODI: Oswestry Disability Index; VAS: Visual Analog Scale for pain

	Patients with Partial or Complete Mesh Detachment	Patients without Partial or Complete Mesh Detachment	p-value
≤ 3 Months	n = 9	n = 121	
ODI	19.1 ± 19.2	20.0 ± 17.5	0.75
VAS Leg	4.4 ± 7.3	21.7 ± 24.3	0.02
VAS Back	22.2 ± 18.6	24.7 ± 20.3	0.84
12+ Months	n = 14^†^	n = 113*	
ODI	23.9 ± 15.7	14.8 ± 16.8	0.02
VAS Leg	32.1 ± 26.1	22.2 ± 27.1	0.08
VAS Back	37.9 ± 24.2	25.5 ± 24.6	0.04
Values represented as: Mean ± Standard Deviation			
^†^One patient had incomplete scores for ODI, VAS Leg/Back; *one patient reported ODI only.			

A radiographic observation of endplate changes, likely due to interaction between the mesh component of the device and the vertebral endplate, was observed in 24 patients (14%). These were observed as large Modic changes on MRI and/or as penetrations of the mesh through the vertebral endplate with associated bone resorption on radiographs. These radiographic changes were without any significant increase in clinical symptoms at either time point versus patients in which this radiographic finding was absent (p = 0.77).

Primary vs. secondary discectomy patients

By 12-month post-op, primary herniation patients exhibited slightly better mean clinical outcome scores than secondary herniation patients, but without statistical significance (p = 0.427; Table 5). No symptomatic reherniations were observed in secondary herniation patients compared to six (4.1%) in the primary herniation group (p = 0.592). Only one reherniation without radicular symptoms (3.9%) was observed in the secondary herniation group versus four (2.8%) in the primary reherniation group (p = 0.566).

**Table 5:** Preoperative and postoperative patient reported outcome scores stratified by primary and secondary herniation at time of index surgery. ODI: Oswestry Disability Index; VAS: Visual Analog Scale for pain.

	Primary Herniation	Secondary Herniation	p-value
Pre-Op	n = 145*	n = 26	
ODI	53.0 ± 21.2	53.0 ± 19.2	0.98
VAS Leg	79.3 ± 23.2	77.7 ± 24.4	0.94
VAS Back	59.5 ± 31.1	55.8 ± 32.3	0.61
≤ 3 Months	n = 111	n = 19	
ODI	20.0 ± 18.0	19.6 ± 15.4	0.90
VAS Leg	20.2 ± 24.4	22.6 ± 21.0	0.42
VAS Back	23.4 ± 19.6	31.1 ± 22.6	0.13
12+ Months	n = 107*	n = 20	
ODI	15.6 ± 17.1	16.7 ± 16.3	0.52
VAS Leg	22.5 ± 26.7	27.5 ± 29.5	0.43
VAS Back	26.3 ± 24.8	30.0 ± 25.1	0.49
Values represented as: Mean ± Standard Deviation			
Symptomatic Reherniation	4.1% (6/145)	0.0% (0/26)	0.59
Reherniation, No radicular pain	2.8% (4/145)	3.9% (1/26)	0.57
Latest Follow-up Time (median, range in days)	403 (25 – 2184)	380, 34 – 1377	0.61
*One subject reported ODI only.			

## Discussion

The objective of this study was to assess the performance of an ACD in large annular defects, in limited discectomy patients in a routine practice setting. Furthermore, in performing this analysis, the authors sought to observe data within a heterogeneous patient setting, offering broader context in which to potentially extrapolate RCT results.

Bouma, et al., which provided the first prospective account of the same ACD in large annular defect patients, reported symptomatic reherniation at 1.5% (1/65) [26]. The reherniation rate observed in the current study was 3.5% for the collective population and 3.2% in the large-defect subpopulation. Of particular note, no symptomatic reherniation occurred in the 26 patients (15.2%) who comprised the secondary herniation group. These rates all compare favorably with the literature in which symptomatic reherniation ranges from 2% to 18% for all defect sizes and varied technique [21, 28]. Furthermore, both the rates observed in Bouma, et al. and the current study are substantially lower compared to those reported by both Kim and Carragee, et al., who in the same large annular defect demographic, reported recurrence rates of 18.0% and 27.3%, respectively, without the use of an ACD [19, 20].

A consideration of the current study not previously examined in the work of Bouma, et al. was that of ACD mesh-related adverse events [26]. As shown in the present study, mesh detachment is possible. However, despite these occurrences, a correlation to complications was unclear. While only two of 15 (13%) patients with mesh detachment underwent a subsequent surgery, significant differences in ODI and VAS Back pain scores existed between patients who experienced detachment and those who did not at follow-up 12 months or greater. Yet, these statistical differences did not result in a level of symptoms that warranted reoperation for the remaining 13 of 15 (87%) patients who experienced mesh detachment. Continued follow-up of patients who experienced mesh detachment but did not undergo subsequent surgery is necessary to further understand any potential pathological cascade that may ensue following such events. Endplate changes were observed within the collective study population (14%). However, the occurrence rate appears to be consistent with a previous report in which endplate changes were observed following lumbar discectomy and found to be without clinical significance at 12 months [29].

Study limitations

The authors acknowledge that there were limitations in this analysis; however, these limitations are partially inherent to registry studies, such as non-standardized follow-up, with no minimum last follow-up. Furthermore, aside from surgical indication and technique, the current study had minimal exclusion criteria, lending to potential confounding demographic or pathological variables. However, the goal of the current study was to assess data with a certain level of heterogeneity in the patient population in order to best represent routine practice setting. An additional limitation was the study being conducted at a single-center on a sample size that may render statistical power insufficiently low for some analyses. While external validity and generalizability may exist between the current study and within this practice alone, consideration must be given to future multi-center registry comparisons.

## Conclusions

The symptomatic reherniation rate of 3.2% among the high-risk patients of this study compares well to historical literature in which large annular defect, limited discectomy patients have exhibited recurrence at much higher rates. The primary differentiator in these outcomes remains the use of ACD augmentation, performing well in both primary and secondary herniation patients. Future comparison of the outcomes presented here to those of an on-going RCT will be important for assessing the generalizability of well-controlled trials.
